# Vascular smooth muscle cell glycocalyx mediates shear stress-induced contractile responses via a Rho kinase (ROCK)-myosin light chain phosphatase (MLCP) pathway

**DOI:** 10.1038/srep42092

**Published:** 2017-02-13

**Authors:** Hongyan Kang, Jiajia Liu, Anqiang Sun, Xiao Liu, Yubo Fan, Xiaoyan Deng

**Affiliations:** 1 Key Laboratory for Biomechanics and Mechanobiology of Ministry of Education, School of Biological Science and Medical Engineering, Beihang University, Beijing, 100191 China

## Abstract

The vascular smooth muscle cells (VSMCs) are exposed to interstitial flow induced shear stress that may be sensed by the surface glycocalyx, a surface layer composed primarily of proteoglycans and glycoproteins, to mediate cell contraction during the myogenic response. We, therefore, attempted to elucidate the signal pathway of the glycocalyx mechanotransduction in shear stress regulated SMC contraction. Human umbilical vein SMCs (HUVSMCs) deprived of serum for 3–4 days were exposed to a step increase (0 to 20 dyn/cm^2^) in shear stress in a parallel plate flow chamber, and reduction in the cell area was quantified as contraction. The expressions of Rho kinase (ROCK) and its downstream signal molecules, the myosin-binding subunit of myosin phosphatase (MYPT) and the myosin light chain 2 (MLC2), were evaluated. Results showed that the exposure of HUVSMCs to shear stress for 30 min induced cell contraction significantly, which was accompanied by ROCK1 up-regulation, re-distribution, as well as MYPT1 and MLC activation. However, these shear induced phenomenon could be completely abolished by heparinase III or Y-27632 pre-treatment. These results indicate shear stress induced VSMC contraction was mediated by cell surface glycocalyx via a ROCK-MLC phosphatase (MLCP) pathway, providing evidence of the glycocalyx mechanotransduction in myogenic response.

Vascular smooth muscle cells (SMCs) normally reside in the tunica media of the arterial wall and play important roles in maintaining and regulating blood vessel tone, blood pressure, and blood flow distribution via cell contraction and relaxation^1^. In the physiological condition, SMCs are shielded from blood flow by an intact endothelium and are not exposed to blood flow induced shear stress directly. However, in the cases of endothelium and internal elastic lamina (IEL) injury, as occur, for example, in angioplasty, near the anastomoses of vascular grafts and in atherosclerotic disease, endothelium disruption or denudation which make SMCs are directly exposed to blood flow^2^. These exposed SMCs will experience blood flow induced shear stress on the order of 10–20 dyn/cm^2^ 3. Even in an intact artery, the underneath SMCs are continuously exposed to a shear stress due to interstitial flow driven by the transmural pressure gradient (typically 100 mmHg in an artery) on the order of 1–3 dyn/cm^2^ 4. Furthermore, it has been predicted by Wang and Tarbell^5^ that the most superficial SMCs, lying directly beneath the IEL, may experience a higher level of shear stress on the order of 10–50 dyn/cm^2^, due to the funneling of flow across the fenestral pores in the IEL.

An *in vitro* work performed by Civelek *et al*.^6^ has shown that rat aortic SMCs in a contractile phenotype, induced by withdrawing serum from normal medium, will exhibit a contractile response when exposed to shear stress *in vitro*. The threshold of shear level for significant shear-induced contraction appeared to be 11 dyn/cm^2^. Moreover, they showed by using the calcium-sensitive fluorescent dye fura 2-AM and pathway inhibitors that this shear-induced contraction was Ca^2+^-independent mainly mediated by the Rho-kinase pathway. This is consistent with the myogenic response (reduction in vessel diameter after an increase in pressure) studies *in vivo* performed by Hill *et al*.^7^. This group pressurized the rat cremaster arterioles (diameter ~100 μm) in the absence of intraluminal flow and examined the Ca^2+^ and diameter responses simultaneously after increases in intravascular pressure were applied as a step increase or a ramp function. The results demonstrated that the acute vessel stretch and associated Ca^2+^ increases are not obligatory for myogenic contraction. Kim *et al*.^8^ hypothesized the fluid shear stress through the vascular wall driven by trans vascular filtration helps regulate arteriolar myogenic tone. They monitored the arteriolar diameter alterations of the rat mesentery before and after vascular occlusion with a glass micropipette and found a statistically significant correlation between the myogenic tone and the fluid filtration rate. Moreover, the myogenic response was attenuated when the fluid filtration was decreased by perfusion the bloodstream with albumin plus Ficoll osmotic solution. These *in vivo* results support the *in vitro* work reported by Civelek *et al*.^6^, indicating the dependence of the myogenic response on the trans vascular fluid filtration and associated shear stress.

More recently, these shear stress-induced SMC contractile responses were further investigated by Ainslie *et al*.^9^ *in vitro*. They exposed the same rat aortic SMCs used by Civelek *et al*.^6^ to a step increase in shear stress and compared the cell contraction rate before and after specific glycosaminoglycans heparan sulfate and chondroitin sulfate removal. Their results indicated that specific components of the SMC glycocalyx may serve as mechano-sensors to regulate shear stress-induced contraction. The cardiovascular cell (EC, SMC, and fibroblast) glycocalyx is a surface layer composed primarily of proteoglycans, with their associated glycosaminoglycan (GAG) that includes heparan sulfate (HS), chondroitin sulfate (CS), and hyaluronic acid (HA), and glycoproteins bearing acidic oligosaccharides with terminal sialic acids^10^. The most common protein backbones of the proteoglycans are syndecans, a transmembrane protein with extracellular and intracellular domains, and glypicans with their global domains terminating in the cell membrane^11^. On the SMC surface, about 50–60% of the GAGs are CS, and the rest are predominantly HS^12^. Although the importance of HS and CS in mechanotransduction of shear stress into SMC contractile responses has been assessed by Ainslie *et al*.^9^, the downstream signaling pathways are still unclear.

It has been well established that the intracellular domains of syndecans, the primary protein backbones of the glycocalyx, have the ability to interact with numerous binding partners and initiate a wide range of signaling networks to regulate diverse physiological processes, including wound healing^13^, arterial development^14^, and blood pressure regulation^15^. In particular, the intracellular domains of syndecans can bind Rho family GTPases like RhoG, Rac1, and RhoA^16^. As molecular switches, these Rho family GTPases orchestrate the remodeling of the actin cytoskeleton and regulate cell motility^17^. Furthermore, as reported by Civelek *et al*.^6^, shear stress induced SMC contraction is mainly mediated by the Rho-kinase pathway that involves RhoA, stimulating Rho-associated kinase (ROCK) then inhibiting myosin light-chain phosphatase (MLCP). The inhibition of MLCP results in an increase in the level of the light chain of myosin (MLC) phosphorylation, which is responsible for regulating SMC contraction^18^.

In the present study, we extended the work performed by Ainslie *et al*.^9^ and investigated the downstream pathways of the glycocalyx mechanotransduction in the regulation of SMC contraction in response to applied shear stress. A parallel plate flow chamber was employed to expose human umbilical cord vein smooth muscle cells (HUVSMC) to a step increase in shear stress (0 to 20 dyn/cm^2^). Reduction in the cell area was quantified as contraction. To determine the role of the glycocalyx in shear-induced SMC contraction, heparinase III (Hep.III) was applied to degrade heparan sulfate selectively from the glycocalyx. Y-27632 was used to inhibit the activity of ROCK. The phosphorylation levels of the downstream signal molecules including the myosin-binding subunit of myosin phosphatase (MYPT) and the myosin light chain 2 (MLC2) were evaluated by Western blot.

## Results

### Contractile phenotype verification

**Figure 1:**
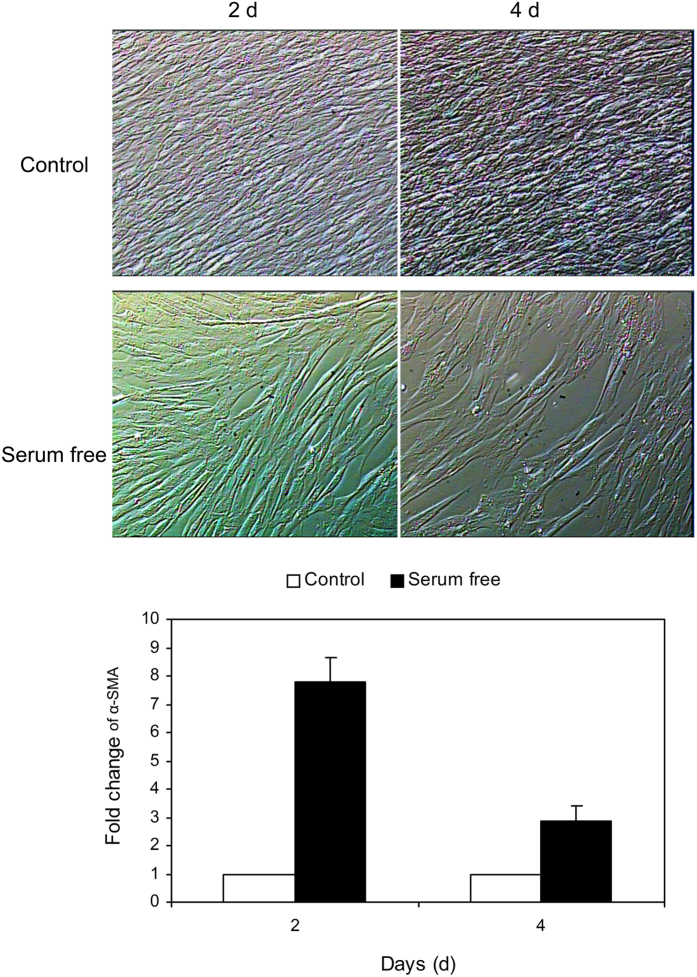
Representative video images of postconfluent HUVSMCs cultured in control smooth muscle cell medium or serum-free DMEM for another 2 and 4 days. The images were taken under ×4 magnification. The mRNA level of smooth muscle а-actin (α-SMA) was measured by real-time PCR and quantified by 2^−ΔΔCt^ method for both control and serum-deprived cells.

**Figure 2:**
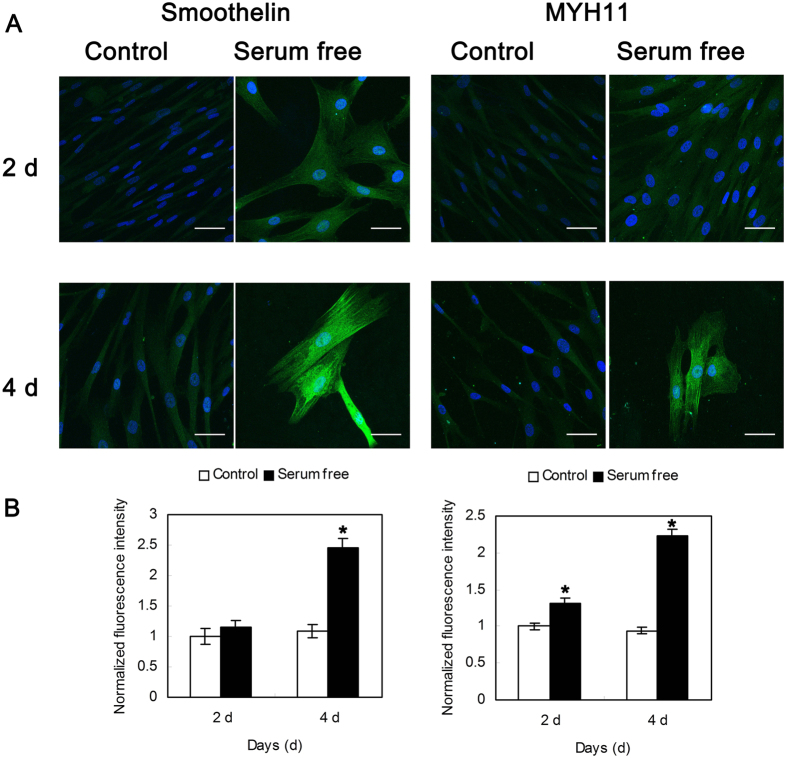
(**A**) Representative immunofluorescence images of smoothelin (left) and MYH11 (right) on the postconfluent HUVSMCs cultured in control smooth muscle cell medium or serum-free DMEM for another 2 and 4 days. Green: smoothelin (left)/MYH11 (right). Blue: DAPI. Scale bar: 50 μm. (**B**) Normalized fluorescence intensity of smoothelin (left) and MYH11 (right). Data were normalized to the control-2 d group. *P < 0.05 vs. Control.

HUVSMCs cultured in SMCM exhibited the characteristic “hill and valley” morphology. To revert confluent HUVSMCs from a synthetic phenotype to a contractile state, serum was deprived from the medium for 2 or 4 days. As shown in Fig. 1, post-confluent HUVSMCs cultured in serum-free medium became elongated and spindle shaped and cannot form multiple layers as the control cells cultured in SMCM. The expression level of contractile marker gene α-SMA showed a respectively 7.80-fold and 2.85-fold increase on post-confluent day 2 and day 4. In addition, the immunofluorescence intensities of smoothelin (Control 4d: 1.09 ± 0.11 vs. serum free 4d: 2.46 ± 0.15, P < 0.05) and MYH11 (Control 2d: 1.00 ± 0.04 vs. serum free 2d: 1.32 ± 0.06, Control 4d: 0.94 ± 0.04 vs. serum free 4d: 2.24 ± 0.08, P < 0.05) were significantly increased after 2–4 day serum starvation of HUVSMCs, as shown in Fig. 2.

### Enzymatic removal of HS

**Figure 3:**
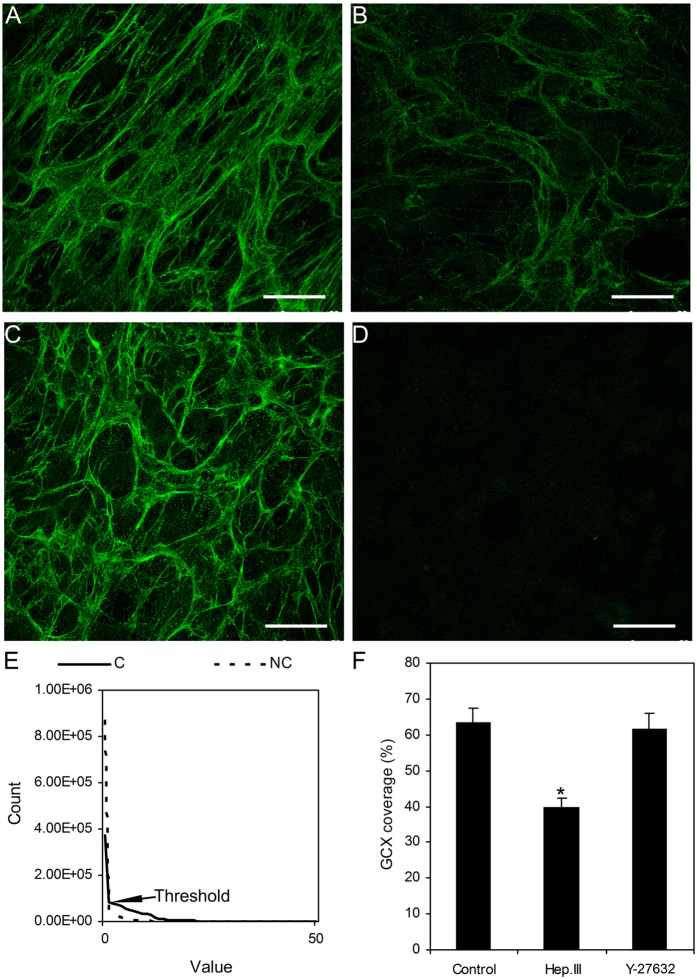
Immunofluorescence images of heparan sulfate proteoglycans on the surfaces of HUVSMCs with and without treatment. (**A**) Control represents without enzymatic treatment. (**B**) 0.2 U/ml Hep.III treated for 1 hour. (**C**) 10 μM Y-27632 treated for 1 hour. (**D**) Negative control. (**E**) The Count-Value curves from the max-intensity Z-projection images of both control and negative control. The non-zero point of intersection between the curves was defined as the background threshold. The coverage of HS was defined as the area with values equal or higher than threshold divided by the total area and presented as a percent. F: The coverage of GCX for control, Hep.III or Y-27632 treated cells. Scale bar: 50 μm. (**C**) Control. NC: Negative control. GCX: glycocalyx. *P < 0.05 vs. control.

Figure 3 shows the presence of HS on the surfaces of cultured HUVSMCs. By using immunofluorescence, we verified the efficiency of Hep.III induced HS cleavage. Approximately 63.7 ± 3.8% of the surface HS was labeled with HS antibody under normal conditions, whereas the coverage of HS was significantly decreased to 39.7 ± 2.6% after 0.2 U/ml Hep.III treatment for 1 hour. It should be noted that the coverage of HS on the surfaces of HUVSMCs treated with 10 μM Y-27632 for 1 hour at 37 °C showed no significant differences as compared to the control group (61.7 ± 4.3%).

### Shear stress induced HUVSMC contraction was mediated by heparan sulfate proteoglycans (HSPG) and ROCK

**Figure 4:**
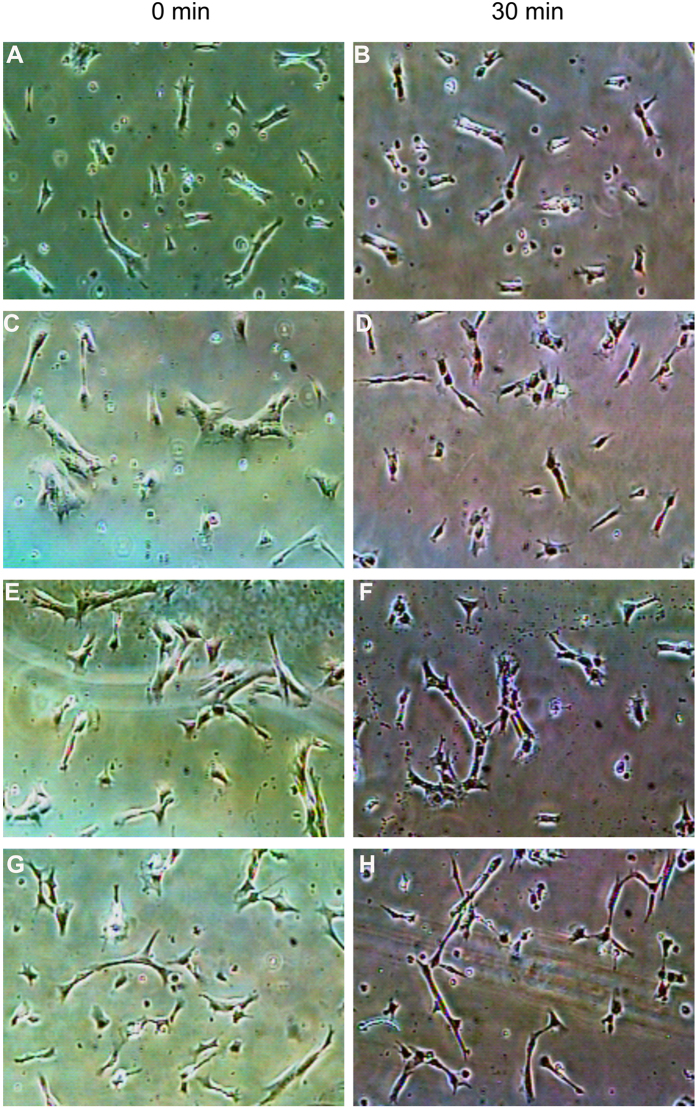
Representative video images of HUVSMCs before (**A**,**C**,**E**,**G**) and after 30-min static (**B**) or 20 dyn/cm^2^ shear stress (**D**,**F**,**G**) exposure. (**A** and **B**) HUVSMCs without enzymatic treatment under static condition. (**C** and **D**) HUVSMCs without enzymatic treatment exposed to shear stress condition. (**E** and **F**) HUVSMCs pre-treated with 0.2 U/ml Hep.III for 1 hour exposed to shear stress condition. (**G** and **H**) HUVSMCs pre-treated with 10 μM Y-27632 for 1 hour exposed to shear stress condition. The images were taken under  x 4 magnification.

**Figure 5:**
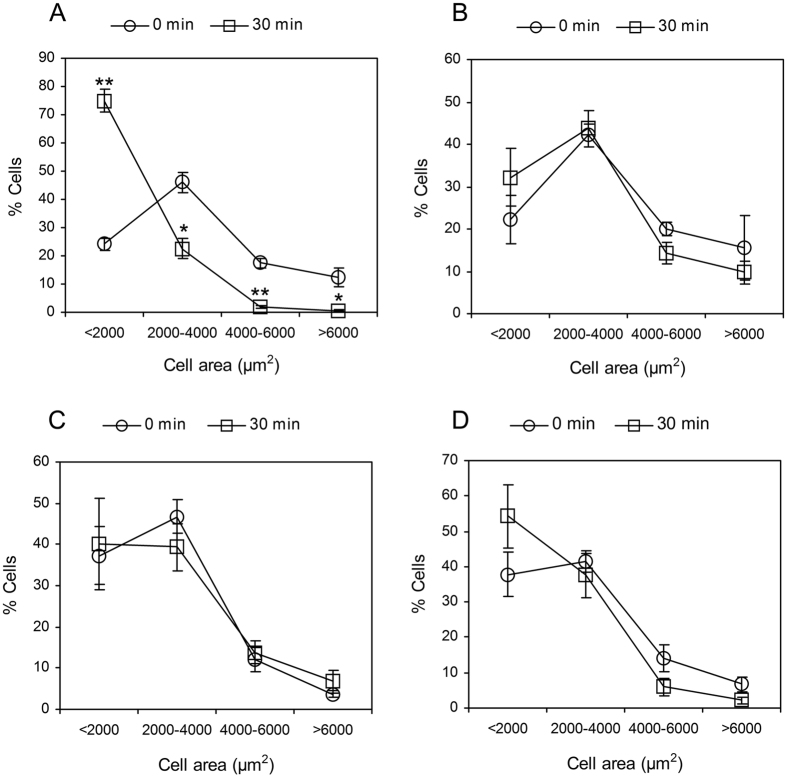
Cell area distributions of HUVSMCs with or without treatment exposed to static or 30-min 20 dyn/cm^2^ shear stress condition. (**A**) Control cells without treatment exposed to shear. (**B**) Control cells without treatment exposed to static. (**C**) HUVSMCs pre-treated with 0.2 U/ml Hep.III for 1 hour exposed to shear. (**D**) HUVSMCs pre-treated with 10 μM Y-27632 for 1 hour exposed to shear. *P < 0.05 vs. time 0. **P < 0.01 vs. time 0.

**Figure 6:**
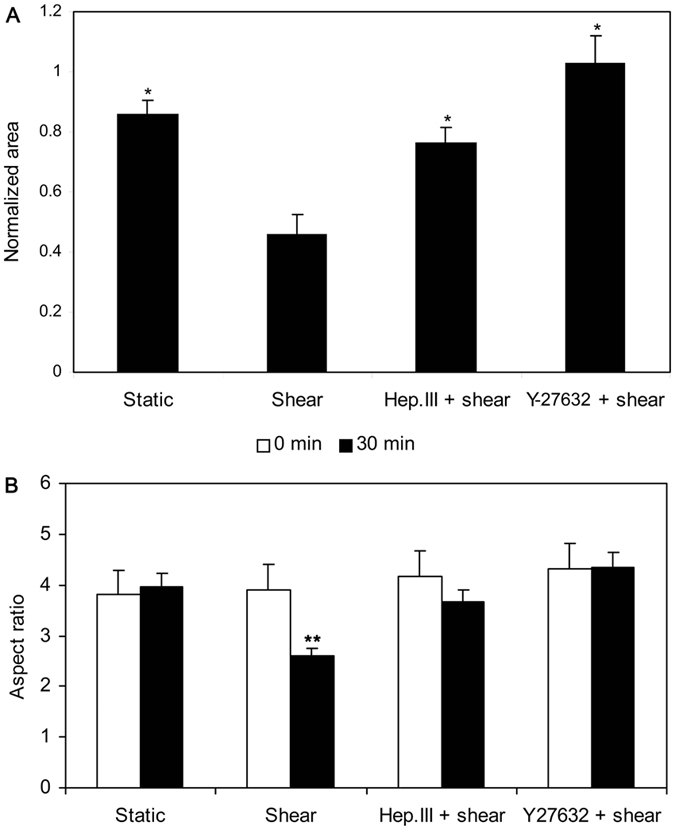
(**A**) Normalized cell area after 30 min static or 20 dyn/cm^2^ shear stress exposure with or without Hep.III or Y-27632 treatment. Cell areas were normalized to the mean value at time 0. (**B**) The aspect ratio of HUVSMCs after 30 min static or 20 dyn/cm^2^ shear stress exposure with or without Hep.III or Y-27632 treatment. 100–300 cells from 5–9 images were analyzed. Data from 4 independent experiments were used to calculate the final mean value ± SEM. *P < 0.05 vs. shear. **P < 0.01 vs. 0 min (Shear).

Figure 4 illustrates a representative video image of HUVSMCs exposed to static or 20 dyn/cm^2^ shear stress. The images were taken under × 4 magnification. The morphology of HUVSMCs starved for 3–4 days before experiments showed some extent of contraction (Fig. 4A and C), and the contraction became more significantly after 30 min-shear stress exposure (Fig. 4D). The area distribution analysis of 100–300 HUVSMCs deprived of serum for 3–4 days, as shown in Fig. 5A, indicated that the initial areas of about 46.0% cells were ranged from 2000 to 4000 μm^2^, the areas of 24.2% cells were smaller than 2000 μm^2^ (<2000), 17.6% cells have the area between 4000 and 6000 μm^2^, only 12.2% cells whose areas were larger than 6000 μm^2^. After 30 min-shear stress exposure, the percent of cells with areas <2000 μm^2^ was increased about 50% (time 0: 24.2 ± 2.2% vs. time 30 min: 75.0 ± 4.0%, P < 0.05), and the cells with areas ranged within 2000–4000 μm^2^, 4000–6000 μm^2^, or >6000 μm^2^ were decreased respectively (Fig. 5A). For the static condition, Cells showed no significant area reduction during the 30-min recording time (Figs 4B and 5B). On the other hand, cells pre-incubated with 0.2 U/ml Hep.III or 10 μM Y-27632 became a little slimmer than the untreated cells (Fig. 4E and G). Area analysis indicated the number of cells with initial areas smaller than 2000 μm^2^ was increased about respectively 13.7% (Hep.III treatment) and 13.1% (Y-27632 treatment) relative to the untreated cells, and shear stress cannot induce significant cell contraction as observed in untreated control cells (Figs 4F,H and 5C,D). Cell area reduction in Fig. 6A illustrated that the exposure of HUVSMCs to 20 dyn/cm^2^ shear stress for 30 min reduced the cell area to 0.46 ± 0.06 fold as compared to the original value (time 0), which is significantly different from the cell area reduction of the static (0.86 ± 0.05 fold), Hep.III (0.76 ± 0.05 fold), and Y-27632 (1.03 ± 0.09 fold) treatment groups. Moreover, the shape factor analysis indicated that there is a significant reduction in the aspect ratio of HUVSMCs exposed to 20 dyn/cm^2^ shear stress for 30 min (0 min: 3.90 ± 0.22 vs. 30 min: 2.60 ± 0.16, P < 0.01), while this significant reduction disappeared after Hep.III (0 min: 4.18 ± 0.36 vs. 30 min: 3.66 ± 0.24, P > 0.24) or Y-27632 (0 min: 4.30 ± 0.30 vs. 30 min: 4.34 ± 0.29, P > 0.93) pre-incubation (Fig. 6B).

### Shear stress induced ROCK1 up-regulation and re-distribution was mediated by heparan sulfate proteoglycans (HSPG)

**Figure 7:**
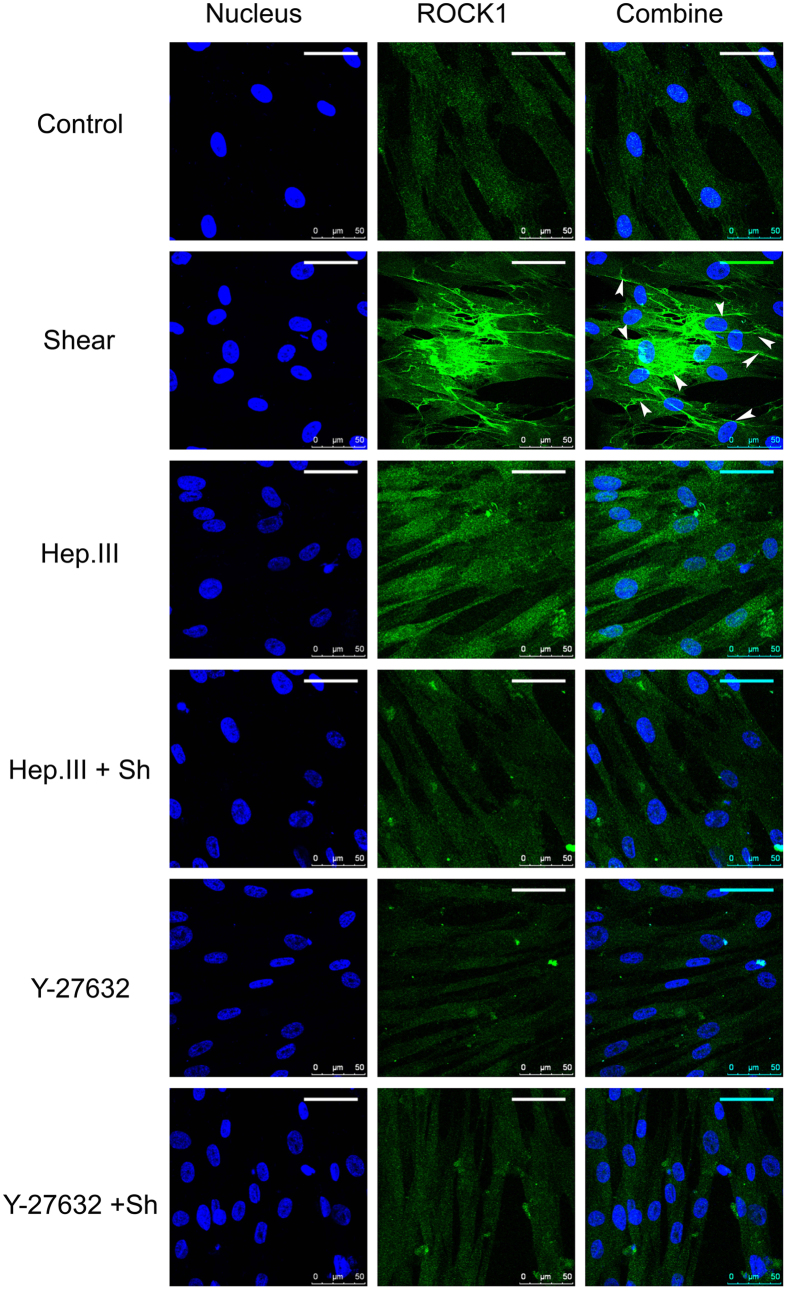
HUVSMCs co-stained with DAPI (nucleus, blue) and ROCK1 antibody (green). Cells were divided into the following 6 groups: control (without treatment and no shear), shear (without treatment but 30-min 20 dyn/cm^2^ shear stress exposure), Hep.III (0.2 U/ml Hep.III treated and no shear), Hep.III + sh (0.2 U/ml Hep.III treated then exposed to shear), Y-27632 (10 μM Y-27632 treated and no shear), and Y-27632 + sh (10 μM Y-27632 treated then exposed to shear). Scale bar: 50 μm. Arrowheads denote ROCK1 distributed on the cell border.

**Figure 8:**
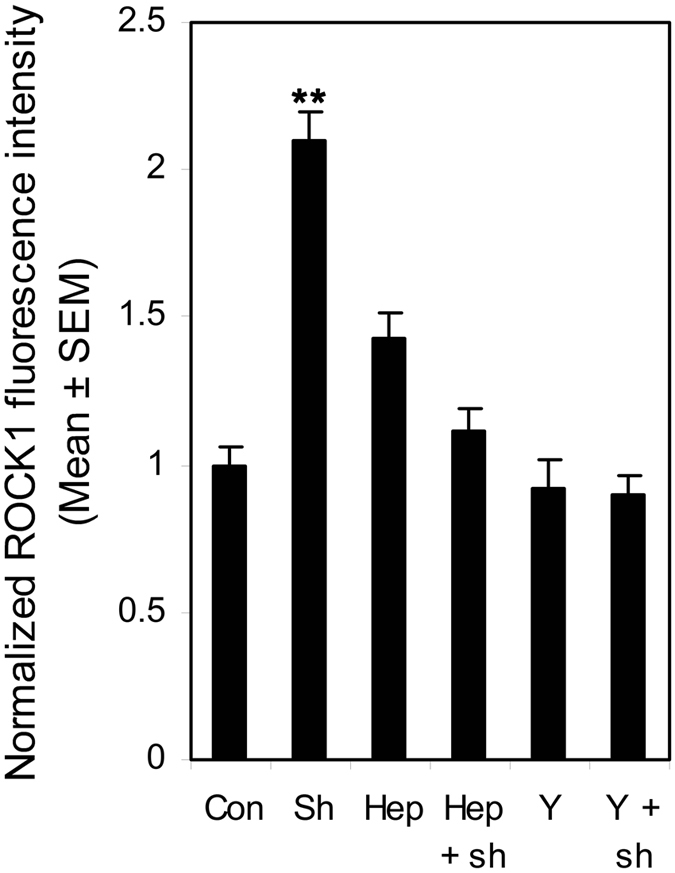
Normalized ROCK1 fluorescence intensity for control and Hep.III or Y-27632 treated HUVSMCs in response to no flow or 30-min 20 dyn/cm^2^ shear stress. The mean fluorescence intensity of ROCK1 was normalized to the control group. Three independent experiments with HUVSMCs from passage 4 to 6 were performed to obtain the final mean value ± SEM. **P < 0.01 vs. control. Con: control cells without treatment and no shear. Sh: control cells without treatment but exposed to 30-min 20 dyn/cm^2^ shear stress. Hep: 0.2 U/ml Hep.III treated and no shear. Hep + sh: 0.2 U/ml Hep.III treated then exposed to shear. Y: 10 μM Y-27632 treated and no shear. Y + sh: 10 μM Y-27632 treated then exposed to shear.

Figure 7 illustrates the representative fluorescence images of ROCK1. Under static conditions, ROCK1 uniformly distributed through the whole cell cytoplasm. After exposing HUVSMCs to 20 dyn/cm^2^ for 30 min, the fluorescence intensity of ROCK1 was increased 2.10 ± 0.10 fold as compared to the static condition (Fig. 8, P < 0.01), and the distribution preferred to concentrating along the cell borders (indicated by arrowheads). Hep.III pre-treatment induced a 1.43 ± 0.09 fold increase of the ROCK1 fluorescence intensity relative to the control group (P > 0.02). On the other hand, Y-27632 pre-treatment induced the ROCK1 fluorescence intensity a little decrease without any statistical significance (Control: 1 ± 0.06 vs. Y-27632: 0.92 ± 0.10, P > 0.05). Interestingly, shear induced ROCK1 re-distribution and fluorescence intensity enhancement were no longer observed in both Hep.III (Hep.III: 1.43 ± 0.09 vs. Hep.III + shear: 1.12 ± 0.07, P > 0.08) and Y-27632 (Y-27632: 0.92 ± 0.10 vs. Y-27632 + shear: 0.90 ± 0.06, P > 0.83) pre-treated cells.

### Shear stress induced activation of ROCK downstream signal molecules MYPT1 and MLC was mediated by heparan sulfate proteoglycans (HSPG)

**Figure 9:**
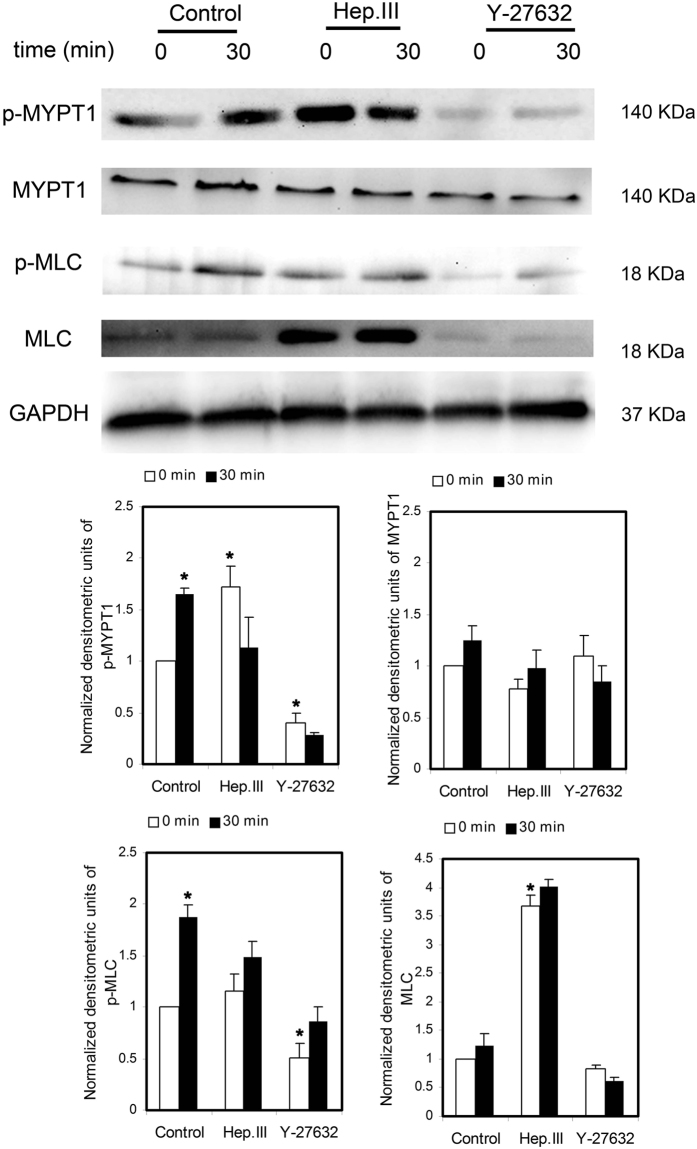
Western blots analysis of MYPT1, p-MYPT1, MLC, and p-MLC of HUVSMCs with or without Hep.III or Y-27632 treatment in response to no flow or 30-min 20 dyn/cm^2^ shear stress. GAPDH was used as the internal reference gene. The densitometry was normalized by GAPDH, then by the control. *P < 0.01 vs. control. For MYPT1, MLC, p-MLC, n = 7; For p-MYPT1, n = 4.

**Figure 10:**
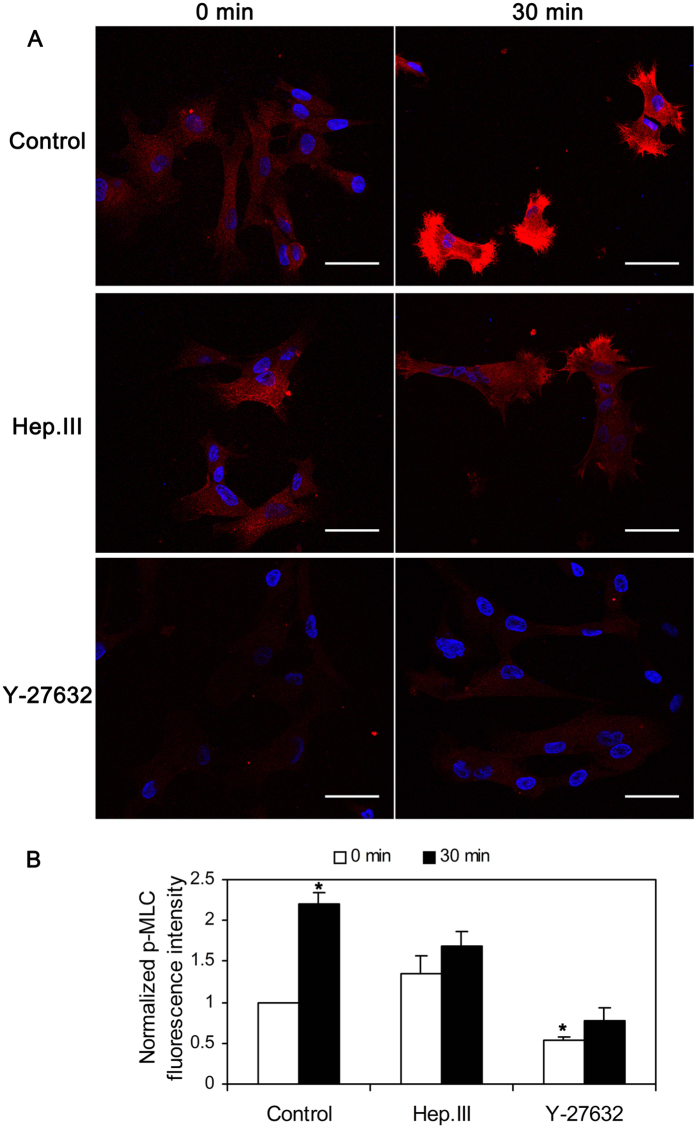
(**A**) HUVSMCs before or after 30-min 20 dyn/cm^2^ shear stress exposure co-stained with DAPI (nucleus, blue) and p-MLC (red). Scale bar: 50 μm. (**B**) Normalized p-MLC fluorescence intensity relative to the control no flow condition. *P < 0.01 vs. control no flow (0 min).

Figure 9 shows representative Western blots and their densitometry for HUVSMCs exposed to no flow control, 30-min shear stress, Hep.III treatment and no flow, Hep.III treatment and 30-min shear stress, Y-27632 treatment and no flow, and Y-27632 treatment and 30-min shear stress. We probed MYPT1, phospho-MYPT1 (Thr853), MLC, phospho-MLC (Ser19), and GAPDH was used as the internal loading control. The densitometric analysis showed that 30 min shear stress exposure of HUVSMCs upregulated the phosphorylation levels of MYPT1 (1.66 ± 0.05 fold) and MLC (1.88 ± 0.12 fold) relative to the no flow control group (P < 0.01), while this up-regulation was completely abolished by Hep.III or Y-27632 treatment. These observations of p-MLC expression were consistent with its fluorescent staining, as we shown in Fig. 10, indicating that 30 min shear exposure will enhance the fluorescence intensity of p-MLC to 2.21 ± 0.13 fold relative to the no flow condition (P < 0.01), and this shear effect could be inhibited when cells were pre-incubated with Hep.III (0 min: 1.35 ± 0.21 vs. 30 min: 1.68 ± 0.18) or Y-27632 (0 min: 0.53 ± 0.05 vs. 30 min: 0.77 ± 0.16). Notably, Hep.III treatment of HUVSMCs under no flow condition per se induced up-regulation of p-MYPT1 (1.72 ± 0.20 fold) and MLC (3.67 ± 0.19 fold) relative to the untreated control cells (P < 0.01). On the other hand, HUVSMCs exposed to Y-27632 before shear stress application showed a significant down-regulation of p-MYPT1 (0.40 ± 0.09 fold) and p-MLC (0.50 ± 0.15 fold) as compared to the untreated control group.

## Discussion

*In vitro* cultured SMCs in serum containing medium are highly proliferative with reduced expressions of SMC contractile marker genes. To revert the dedifferentiated SMCs to contractile state, Civelek *et al*.^6^ and Ainslie *et al*.^9^ maintained rat aortic smooth muscle cells (RASMCs) in serum-free media for 4–7 days and observed 73% starved cells stained positively for the contractile marker smooth muscle α-actin (α-SMA). More earlier, Tagami *et al*.^19^ even detected the spontaneous contractions of RASMCs cultured in serum-free medium for 7–8 days and concluded that SMCs cultured 7–8 days without serum can completely differentiated as *in vivo*. Therefore, in the present study, we used the same serum-withdraw method to induce cultured HUVSMCs into a contractile phenotype. Our results in Fig. 1 showed that after depriving serum for 2–4 days, HUVSMCs became elongated and spindle shaped, meanwhile the expression level of smooth muscle α-actin (α-SMA) was significantly enhanced as compared to the cells cultured in the normal medium. These are consistent with the observations from Han *et al*.^20^ and Yang *et al*.^21^ in different papers that human VSMCs can redifferentiated after 24 h serum deprivation and this redifferentiation became completely by 72 h. Meanwhile, the expression of SMC contractile marker genes reached peak by 48–72 h serum withdraw. In the present study, we starved HUVSMCs for 3–4 days before shear application, which is consistent with the peak differentiation state reported by Han *et al*.^20^. It should be noted that, HUVSMCs deprived of serum for >5 days began to die and shed. However, it should be mentioned that serum deprivation has also reduced the adhesive ability of HUVSMCs under both static and flow conditions (data not shown). This phenomenon may be attributed to serum starvation induced cellular metabolism disruption^22^ including reduced proteins synthesis that was needed by the cells to stick to the plastic surface or mechanical properties alteration^23^. However, this cannot affect the contraction we observed in the present study. The contraction we determined in the present study was the area reduction of HUVSMCs before or after 30 min shear exposure, which was quantified as the area after flow divided by the area before flow for one individual cell, so it was independent of the adhesive cell numbers since we just calculated the area alteration of the reliquus cells after flow application.

It has been demonstrated previously that heparinase has the ability to alter not only the thickness^24^ but also the barrier property of the glycocalyx^25^. Ainslie *et al*.^9^ reported that 0.2 U/ml heparinase treatment of RASMCs for 30 min induced a 38% reduction in heparan sulfate fluorescence intensity but no significant depletion of the chondroitin sulfate, which substantiated the specificity and effectiveness of this enzyme. Our previous studies^26^ by exposing RASMCs to the same concentration of heparinse obtained very similar HS depletion efficiency (39.67 ± 1.30%) as Ainslie *et al*. reported. In the present study, we exposed HUVSMCs to 0.2 U/ml Hep.III (the same concentration as Ainslie *et al*. used) and observed an approximate 24% reduction in the coverage of heparan sulfate proteoglycans, which is much close to the value obtained by Ebong *et al*.^27^ in bovine aortic endothelial cells (BAEC). They treated BAECs with 15 mU/mL of HepIII for 2 hours and observed the HS coverage reduced from 67% (before treatment) to 46% (after treatment). Again, our fluorescence results verified the effectiveness of HS removal.

The cell area reduction has been widely used to measure the contractile state of SMCs. By using this method, Civelek *et al*. performed the first *in vitro* study of SMC contraction in response to well controlled shear stress and measured the area reduction was 30.8 ± 1.4% after 30 min 25 dyn/cm^2^ exposure, which provided indirect evidence suggesting that except for stretch and tension, the interstitial fluid shear stress on SMCs may be another mechanical force mediating myogenic response. More recently, the studies of Ainslie *et al*. measured the normalized area response of RASMC at the 30-min time point were 89.3 ± 3.8% (Control), 81.1 ± 3.0% (HP Shear), and 61.4 ± 3.3% (25 dyn/cm^2^), indicating that specific components of the SMC glycocalyx, heparan sulfate, play an important role in the mechanotransduction of shear stress into a contractile response. Consistent with those studies, we reported the cell area of HUVSMCs after 30-min 20 dyn/cm^2^ exposure were 0.86 ± 0.05 fold (static), 0.46 ± 0.06 fold (shear), 0.76 ± 0.05 fold (Hep.III) relative to the time 0 point. In addition, we also observed that after incubating the cells with Y-27632, a specific ROCK inhibitor, shear-induced SMC contraction was completely abolished. The measured area at 30-min time point was 1.03 ± 0.09 fold, which was consistent with the results reported by Civilek *et al*. They pre-incubated RASMCs with 10 μg/ml exoenzyme C3 (an inhibitor of Rho kinase pathway) for 4 hours and observed a suppression of contraction induced by shear. Although two different inhibitors were used, Y-27632 vs. exoenzyme C3, we and Civelek *et al*. all observed the evidence that Rho-kinase pathway may be the most important signal transduction pathway mediating SMC contraction in response to shear stress.

The fluorescence images of ROCK1 in Fig. 7 showed a uniform distribution through the whole cell cytoplasm, which is consistent with the confocal images from bovine tracheal smooth muscle obtained by Sommer *et al*.^28^. They co-stained the bovine tracheal smooth muscle cryosections with ROCK1 and Cav-1 antibodies and observed the distribution of ROCK1 throughout the cytosol as well as in the cell membrane, but no colocalization with Cav-1. Moreover, shear stress modulated RhoA/Rho kinase system was systematically studied by Wesselman *et al*.^29^. By using cDNA microarray analysis, they quantified the differential expression of >14,000 genes from rat mesenteric small arteries that have been subjected to flow-modifying surgery and demonstrated the importance of the RhoA/Rho kinase system in flow-related small artery remodeling. Consistent with Wesselman’s study, we also observed shear stress- induced ROCK1 translocation (Fig. 7) and upregulation (Fig. 8) in cultured HUVSMCs. In addition, the phosphorylation of ROCK downstream signal molecules MYPT1 and MLC were all enhanced (Fig. 9), and the above mentioned ROCK upregulation and activation were completely abolished when HUVSMCs were pre-incubated with 10 μM Y-27632 for 1 hour before shear application. As we have suggested before, herein shear stress induced ROCK and its downstream molecules activation provided more direct evidence that Rho-kinase pathway indeed participated in the process of mediating SMC contraction in response to shear stress.

It should be noted that phosphorylated MYPT1 and MLC were clearly detectable in control starved cells without flow in the present study (Fig. 9). This phenomenon is consistent with some other previously reported studies. Hudson *et al*.^30^ isolated smooth muscle cells from myometrial tissue and evaluated the effect of ROCK inhibitor g-H-1152 on the phosphorylation of MLC and MYPT1 after 4 h serum starvation of the SMC confluence. Results indicated clearly detectable bands of pMYPT1 (T853) and phosphorylted myosin light chains (pMYL) in control cells without g-H-1152 treatment. Moreover, the detectable phosphorylated MLC and MYPT1 were also reported by Wang *et al*.^31^ in cultured human vascular smooth muscle cells, attempting to elucidate the effects and molecular mechanisms of AMP-activated protein kinase (AMPK) regulated smooth muscle contraction and blood pressure in mice. Bjork *et al*.^32^ have observed myosin light chain phosphorylation in cultured A7r5 smooth muscle cells from rat aorta after serum starvation with or without inhibitor (dexmedetomidine) preincubation, which is consistent with the observations from Zhang *et al*.^33^ that 18 h-serum starvation of A7r5 cells phosphorylated MLC as well.

On the other hand, shear induced ROCK1 re-distribution, upregulation, and activation could also be inhibited by Hep.III treatment, which suggests the key upstream mechanotransduction role of heparan sulfate proteoglycans in shear stress induced SMC contraction, as Ainslie *et al*.^9^ has reported before. However, it should be noted that, Hep.III pre-treatment per se, induced an increase in p-MYPT1 (1.72 ± 0.20 fold) and MLC (3.67 ± 0.19 fold) expression relative to the control group. This is beyond our expectation. The possible mechanism may be that the interaction between cell surface heparan sulfate proteoglycans (HSPG) and the heparin-binding domains of fibronectin is necessary for focal adhesion formation^34^ to regulate cell migration, adhesion and contraction^35^, which is also RhoA-ROCK pathway associated^36^. Once the cell surface heparan sulfate was cleaved by heparinase, the excess soluble heparan sulfates would act as the competitor of heparin-binding domains of fibronectin^37^, then adhesion formation may be inhibited and cell migration^26^,^38^ or contraction may be facilitated. During these processes, ROCK may be activated to some extent. However, this is just our speculation and there is much future work needed to be carried out.

In conclusion, the present study extended the previous work performed by Ainslie *et al*., attempting to elucidate the downstream pathways of the glycocalyx mechanotransduction in the regulation of SMC contraction. We have shown that the exposure of HUVSMCs to 20 dyn/cm^2^ shear stress for 30 min reduced the cell area to 0.46 ± 0.06 fold as compared to its original area value (time 0), induced ROCK1 up-regulation (2.10 ± 0.10 fold), re-distribution (concentrating along the cell borders), as well as ROCK downstream molecules MYPT1 and MLC activation. However, these shear induced cell contraction (cell area reduction), ROCK and its downstream signal molecules activation could be completely abolished by Hep.III or Y-27632 (a specific ROCK inhibitor) pre-treatment. These results suggest that shear stress induced vascular smooth muscle cell contraction was mediated by cell surface glycocalyx via a RhoA-Rho kinase (ROCK)-myosin light-chain phosphatase (MLCP) pathway, which provided evidence suggesting the importance of the vascular smooth muscle cell glycocalyx mechanotransduction in the myogenic response.

## Material and Methods

### HUVSMC culture

Smooth muscle cells were obtained from human umbilical cord veins provided by Haidian Maternal & Child Health Hospital (Beijing, China). The protocol was approved by the Institutional Committee for the Protection of Human Subjects of Beihang University and informed consent was obtained from all subjects. All experiments were performed in accordance with the relevant guidelines and regulations. In brief, after isolation from the placenta, the cord was kept at sterilized phosphate buffer solution (PBS) and stored at 4 °C for use. The endothelial cells were removed by injecting a digestion solution (2.5 mg/mL trypsin and 1 mg/mL collagenase II) into the vein and keeping it at 37 °C for 10 min. After harvesting endothelial cells, the umbilical cord was cut open longitudinally, split into pieces (~5 mm), and placed onto a T-25 culture flask with the luminal surface of the explant down. The explants were kept stationary in the incubator for about 2 hours to make its luminal surface adhere to the culture flask tightly, then a 5 ml smooth muscle cell medium (SMCM, ScienCell research laboratories, CA) was added. Cells were identified as human umbilical vein smooth muscle cells (HUVSMCs) by staining positively for a-smooth muscle actin, negatively for von Willebrand factor, and displaying typical ‘hill and valley’ morphology when confluent. We used cells between passages 2 and 6.

### Contractile HUVSMC verification

The contractile phenotype of HUVSMCs was induced by starving the cell monolayers of fetal bovine serum (FBS) in DMEM (high glucose, Invitrogen, Camarillo, CA) supplemented with 100 U/ml penicillin and 100 μg/ml streptomycin (1% P/S) for 2 days. The cells then were detached from the flask with 0.25% trypsin, seeded on the 25.4 × 76.2-mm glass slides precoated with 1 μg/ml fibronectin (BD Biosciences, San Jose, CA) with DMEM plus 1% P/S at a density of 1.0–2.5 × 10^5^ cells/slide, and starved for additional 1–2 days before experiments.

The contractile phenotype of HUVSMCs was verified by their expression of smooth muscle а-actin (α-SMA). The amount of total RNA from control and serum-starved cells were quantified with a NanoDrop 2000 spectrophotometer (Thermo Scientific, Delaware, USA). RNA was subsequently reverse transcribed using PrimeScriptTM RT reagent Kit (Takara Bio INC., Otsu, Japan) and Real-time PCR was performed on iQ5 Multicolor Real Time PCR Detection System (Bio-Rad, Hercules, CA) using SYBR Green master mix (Takara Bio INC., Otsu, Japan). The forward primer employed for α-SMA is 5′-GACAGCTACGTGGGTGACGAA-3′, and the reverse primer is 5′-TTTTCCATGTCGTCCCAGTTG-3′. Quantification of the relative changes in mRNA levels between control and serum-starved cells was performed using the delta delta threshold cycle (ΔΔCt) method. ΔCt was calculated by subtracting the Ct value of the α-SMA from the Ct value of the internal reference gene GAPDH. ΔΔCt was obtained by subtracting the ΔCt value of the serum-starved group from the ΔCt value of the control group in the same experiment. Fold change was then calculated as 2^−ΔΔCt^. In addition, the immunofluorecence staining of smoothelin and MYH11 was performed after 2–4 day serum starvation of HUVSMC.

### Shear apparatus

A parallel plate flow chamber developed in our own laboratory was used to expose cultured HUVSMC monolayers to laminar fluid shear stress as we described elsewhere^26^. Briefly, the flow chamber was made by sandwiching a silicone gasket between the glass slide and a Plexiglas plate. Fluid circuit was comprised of a peristaltic pump (Longer pump, Hebei, China), a flow chamber, a pulse dampener (Cole-Parmer China, Shanghai, China), and two reservoirs with air filters on their caps. Silicone tubes and 3-way stopcocks were used to connect each part and regulate the flow. Shear stress was determined by the following equation: τ = 6 μQ/Wh^2^, where τ is shear stress (dyn/cm^2^), μ is viscosity of the medium (0.0078 poise), Q is the flow rate across the flow chamber (ml/s), h is channel height (500 μm for standard gasket), and W is channel width (3.8 cm for our chamber). During the flow experiments, the flow system was kept at 37 °C 5% CO_2_ incubator. All experiments were conducted at 20 dyn/cm^2^. This level of shear stress is high enough to induce significant contraction of SMCs as reported by Civelek *et al*.^6^.

### SMC contraction analysis

Cells before or after 30 min-shear exposure were captured by an invert microscope (Olympus Optical, Japan) equipped with a charge-coupled device camera (TK-C1381, JVC, Tokyo, Japan) and a computer with a frame grabber. Images were imported and analyzed by using Image J software (National Institute of Health, USA) that is free and available from the website. After being scaled, images with clear cell outlines and low background were chosen to calculate the cell areas. The outlines of cells were traced manually by using the polygon selections tool and the individual cell area was calculated by the software. 5 to 9 images from each time point were analyzed and areas of 100–300 cells were calculated for each experiment. The areas of cells were divided into 4 levels including small than 2000 μm^2^ (<2000), 2000–4000 μm^2^, 4000–6000 μm^2^, and larger than 6000 μm^2^ (>6000). The distribution of cell areas before or after shear exposure was determined by the number of cells within each level divided by the total number of cells and presented as a percent. Reduction in cell area before or after 30 min shear exposure was measured and used as a criterion for contraction. The mean area of 100–300 cells from 5–9 images at 30 min was normalized with respect to the mean value at 0 min. Moreover, the aspect ratio of HUVSMCs was analyzed by the “shape descriptors” of Image J in order to describe the shape alterations before and after shear exposure. Data from 4 independent experiments were used to calculate the final mean value ± SEM.

### Heparinase III treatment

Heparinase III (Hep.III) from Flavobacterium heparinum (Sigma-aldrich, St. Louis, MO) was applied to cleave the specific components of the SMC’s glycocalyx. The enzyme was reconstituted in 20 mM Tris-HCl, pH 7.5, containing 0.1 mg/ml BSA and 4 mM CaCl_2_, aliquoted, and stored at −20 °C. It was freshly diluted in DMEM + 1% P/S and used at a concentration of 0.2 U/ml. The conflunent monolayers on glass slides were treated with this enzyme for 1 hour in a Petri dish inside a 95% air and 5% CO_2_, 37 °C incubator. Before mounting on the flow chamber, the glass slides were washed with fresh DMEM + 1% P/S twice.

### Y-27632 treatment

To inhibit the activity of Rho kinase, cells were incubated in Y-27632 dihydrochloride (abcam Bochemicals, Cambridge, UK) at a final concentration of 10 μM in DMEM + 1% P/S for 1 hour at 37 °C. Before mounting on the flow chamber, the glass slides were washed with fresh DMEM + 1% P/S twice.

### Inmmunofluorescence and confocal microscopy

Immunofluorescence staining of HS, ROCK 1, and p-MLC was performed by using a protocol as we described before. Briefly, the HUVSMC monolayers was washed quickly with PBS and fixed with 4% paraformaldehyde for 10 min. For the ROCK1 and p-MLC staining, cells were perrmeabilized with 0.2% triton X-100 in PBS for 10 min, while for HS staining, this permeabilization was omitted. After washing in PBS, cells were blocked with 10% goat serum for 1 hour at room temperature followed by an overnight incubation with the primary antibody anti-heparan sulfate (1:100, F58–10E4, amsbio, Cambridge, MA), anti-ROCK1 antibody (1:100, ab45171, abcam, Cambridge, UK), or phospho-myosin light chain 2 Ser19 mouse antibody (1:200, 3675, Cell Signaling Technology, Danvers, MA) at 4 °C. To gain fluorescent label, monolayers were then incubated with Alexa Fluor 488 goat anti-mouse IgM (1:400, Molecular Probes, USA), Alexa Fluor 488 goat anti-rabbit IgG (1:200, abcam, Cambridge, UK), or rhodamine conjugated goat anti mouse IgG (1:200, Santa Cruz Biotechnology, CA) for 30 min at room temperature in dark. After washing in PBS three times with 5 min interval, the slides were mounted with Antifade mounting medium with DAPI (HelixGen, Guangzhou, China) and imaged under a Leica TCS SPE confocal microscope (Leica Microsystems, Wetzlar, Germany). A ×40 oil objective lens was applied and the field of view (FOV) was about 275 μm × 275 μm with a 1,024 × 1,024 pixel solution. The interval between slices was set at 0.3–0.5 μm for a Z-series stack scanning. For each slide, 6 FOVs were chosen and three slides from independent experiments were prepared for both control and Hep.III treatment groups.

Images were analyzed by using Image J software (National Institute of Health, USA) and the coverage of HS was quantified as Zeng *et al*. described^24^. In brief, the maximum intensity Z projection of the green Z-series stack (Alexa Fluor 488 channel) was created, in which each pixel contains the maximum value over all images in the stack at the particular pixel location. The pixel intensity histograms from the FOV of maximum intensity Z projection images of both control and negative control were obtained by using the histogram tool. Mean Counts at each value from 6 FOVs for both control and negative control were calculated and the Count-Value curves were obtained. The point of intersection was defined as the threshold. The coverage of HS was defined as the area with values equal or higher than threshold divided by the total area of FOV and presented as a percent. The mean fluorescence intensity of ROCK1 was quantified and normalized to the control group.

### Western Blot

HUVSMC monolayers were lysed in 200 μL RIPA Lysis Buffer supplemented with Protease Inhibitor Cocktail (1 tablet in 10 ml lysis buffer, Roche, Indianapolis, IN) and PhosSTOP (1 tablet in 10 ml lysis buffer, Roche, Indianapolis, IN) on ice for 30 min and centrifuged at 14,000 g for 10 min. Protein concentration in the supernatant was determined by an Enhanced BCA Protein Assay Kit (Beyotime, Jiangsu, China) according to the manufacturer’s instructions. 50 μg of total protein were mixed with 5 × SDS loading buffer, boiled for 10 min, and separated in denaturing SDS/12.0% polyacrylamide gels. Thereafter, proteins were transferred to PVDF membranes (Millipore, Bedford, MA) and blocked for 90 min at room temperature with 5% nonfat dry milk in TBS-T. The membranes were then incubated with primary antibodies against MYPT1 (diluted at 1:1000), phospho-MYPT1 Thr859 (diluted at 1:1000), myosin light chain 2 (diluted at 1:1000), phospho-myosin light chain 2 Ser19 (diluted at 1:1000) and GAPDH (diluted at 1:500) overnight at 4 °C. After washing in TBS-T three times with 10 min interval, the membranes were incubated in ECL horseradish peroxidase–conjugated secondary antibodies (diluted at 1:5000) for 1.5 h at room temperature followed by washing three times in TBS-T. The proteins on PVDF membranes were then detected by using Efficient chemiluminescence kit (GENVIEW, Calimesa, CA) and the SmartChemi^TM^ Image Analysis System (Sagecreation, Beijing, China). Each experiment was repeated at least three times.

### Statistics

Data are presented as mean ± SEM. D’Agostino & Pearson omnibus normality test was performed to test if the values come from a Gaussian distribution. Differences were assessed by using unpaired Student’s t-test, Ratio paired t test, or Wilcoxon matched-pairs signed rank test when small numbers are compared or data are non-normally distributed with P < 0.05 considered significant. For multiple comparisons, the Bonferroni correction was used, which gives a conservative significance level of P divided by the number of comparisons. Each experiment were repeated three or more times.

## Additional Information

**How to cite this article**: Kang, H. *et al*. Vascular smooth muscle cell glycocalyx mediates shear stress-induced contractile responses via a Rho kinase (ROCK)-myosin light chain phosphatase (MLCP) pathway. *Sci. Rep.* **7**, 42092; doi: 10.1038/srep42092 (2017).

**Publisher's note:** Springer Nature remains neutral with regard to jurisdictional claims in published maps and institutional affiliations.
